# OSI-027 as a Potential Drug Candidate Targeting Upregulated Hub Protein TAF1 in Potential Mechanism of Sinonasal Squamous Cell Carcinoma: Insights from Proteomics and Molecular Docking

**DOI:** 10.3390/biology13121089

**Published:** 2024-12-23

**Authors:** Watcharapong Panthong, Chamsai Pientong, Thawaree Nukpook, Chukkris Heawchaiyaphum, Sirinart Aromseree, Tipaya Ekalaksananan

**Affiliations:** 1 Department of Microbiology, Faculty of Medicine, Khon Kaen University, Khon Kaen 40002, Thailand; watchara.p@kkumail.com (W.P.); thawaree.n@kkumail.com (T.N.); chukhe@kku.ac.th (C.H.); sirinar@kku.ac.th (S.A.); 2 HPV&EBV and Carcinogenesis (HEC) Research Group, Faculty of Medicine, Khon Kaen University, Khon Kaen 40002, Thailand

**Keywords:** sinonasal squamous cell carcinoma, proteomic, spliceosome, repurposing drug, OSI-027

## Abstract

Sinonasal squamous cell carcinoma (SNSCC) is a rare tumor with high mortality and recurrence rates. However, SNSCC carcinogenesis mechanisms and potential therapeutic drugs have not been fully elucidated. This study investigated the key molecular mechanisms and hub proteins involved in SNSCC carcinogenesis using proteomics and bioinformatic analysis. Dysregulated proteins were validated by RT-qPCR in SNSCC and nasal polyp (NP) tissues. Proteomic analysis revealed that differentially expressed proteins were clustered using MCODE scores ≥ 4 into three modules. The specific hub proteins in each module were analyzed in carcinogenesis pathways using STRING, highlighting potential mechanisms of histone modification and spliceosome dysregulation. Spliceosome components SNRNP200 and SF3A3 were significantly downregulated in SNSCC by RT-qPCR. Web-based applications L1000CDS^2^ and iLINCS were applied to identify 10 potential repurposable drugs that could reverse the gene expression pattern associated with SNSCC. Docking studies of TAF1, a protein in histone modification, with these 10 small molecule inhibitors indicated OSI-027 to be the most promising due to its strong binding interactions with key residues. These findings suggest that hub proteins involved in the underlying mechanism of SNSCC carcinogenesis may serve as valuable targets for drug development, with OSI-027 emerging as a novel candidate against TAF1 in SNSCC.

## Simple Summary

Sinonasal squamous cell carcinoma (SNSCC) has a high mortality rate and lacks targeted therapy options. This study explored the molecular mechanisms underlying SNSCC carcinogenesis, hub proteins, and drug therapeutic targets using an integrative analysis of proteomics and bioinformatics approaches. Protein expressions were clustered into three modules, emphasizing histone modification and spliceosome dysregulation. The spliceosome components SNRNP200 and SF3A3 were significantly downregulated in SNSCC, as evidenced by RT-qPCR. Among the ten candidate repurposable drugs identified, molecular docking demonstrated OSI-027 to be a promising therapeutic drug for SNSCC, targeting TAF1. These findings highlight the potential mechanism of SNSCC carcinogenesis and OSI-027, a potential drug against TAF1 in SNSCC.

## 1. Introduction

Sinonasal cancers account for approximately 5% of all head and neck cancers, with an annual global incidence of approximately one case per 100,000 people [1,2,3]. Malignant sinonasal tumors are a diverse group, with sinonasal squamous cell carcinoma (SNSCC) being the most common at 50%, followed by intestinal-type adenocarcinoma (ITAC) at 13%, among others [4]. SNSCC, which typically arises in the nasal cavity and maxillary sinus, often presents with non-specific symptoms such as nasal blockage, facial pain, runny nose, and nosebleeds [5]. Consequently, SNSCC is frequently diagnosed at an advanced stage, contributing to a poor prognosis and high morbidity from treatment. The 5-year overall survival rate for SNSCC is about 50–60%, with a recurrence rate of 20–30% also leading to a poor survival rate [4,6]. However, the exact cause of SNSCC is not fully understood.

Occupational factors play a significant role in the development of SNSCC, which may help explain its high prevalence in males. Exposure to industrial compounds and chemical substances, such as leather dust, chrome, nickel, welding fumes, arsenic, and textiles, accounts for up to 10% of SNSCC cases [3,7,8].

Genetic mutations and oncogenic viruses are associated with the development of SNSCC. An in vitro model study from our group linked EGFR mutation to the development of sinonasal inverted papilloma (SIP) from nasal polyps (NP) [9]. Nasal polyp cells expressing EGFR exon 20 duplication mutants S768_D770dup and N771_H773dup exhibited an inverted growth pattern in a 3D organotypic raft culture model. These two types of mutations have been reported to be resistant to EGFR inhibitors (erlotinib and gefitinib) in SIP-associated SNSCC cell lines [10]. Moreover, SIP can undergo malignant transformation in up to 13% of cases [11,12,13]. EGFR mutations in exon 20 are the most frequently observed mutations in SIP and SIP-associated SNSCC in up to 88% and 77% of cases, respectively, indicating that EGFR mutations are early events driving SIP development and SIP-associated SNSCC [10,14]. A high frequency of mutated KRAS is also found in both oncocytic sinonasal papilloma (OSP) and OSP-associated SNSCC [14,15]. In addition, mutations of TP53 and CDKN2A have been implicated in the malignant progression to sinonasal carcinoma [16]. Overall, genetic mutations and environmental factors significantly influence SNSCC carcinogenesis.

Human papillomavirus (HPV) and Epstein–Barr virus (EBV) have been found to be involved in SNSCC. In a systematic review, HPV was identified in 30% of all cases of SNSCC [17]. Among the HPV genotypes detected, HPV-16 has the highest prevalence, followed by HPV-18 and -33, either in singular infections or in co-infection with other HPV genotypes [18,19,20]. In the histopathological classification of SIP and SNSCC, EBV infection is mainly found in undifferentiated and poorly differentiated SNSCC cases, as well as in SIP cases with mild dysplasia [21]. Recent studies have reported a high prevalence of EBV (45.5%) in SNSCC, with EBV-positive cases associated with an increased rate of lymph node or distant metastases [22]. Therefore, the presence of HPV and EBV in SNSCC tissues may be a significant risk factor for disease progression.

Proteomic and bioinformatic analyses have been developed for identifying protein expression profiles associated with pathophysiological conditions [23]. The method offers insights into underlying molecular mechanisms, the biological consequences of altered gene expression, and potential biomarkers [23,24]. With this method, researchers have identified key hub proteins with significant clinical implications for cancer development and treatment [25]. For instance, ERO1A and FEN1 have been proposed as prognostic markers for lung adenocarcinoma [26]. SERPINH1 has been suggested as both a prognostic indicator and a potential contributor to the development of colorectal cancer [27].

Most SNSCC patients are diagnosed at advanced stages, with tumors invading critical structures such as the orbit or skull base, making treatment challenging. The advanced stage of SNSCC often require multimodal therapy, including surgery and radiotherapy, to improve local tumor control and overall survival rates [28]. Currently, targeted therapy options for malignant sinonasal tumors are lacking. The identification of inhibitors targeting hub proteins could address this gap. Recently, molecular docking has become a critical tool for computer-assisted drug design. This method offers a rapid and cost-effective way to identify small-molecule compounds that are against hub protein [29]. Additionally, this strategy could support personalized medicine by using existing drugs for new therapeutic uses or repurposing drugs for specific patients, considering their unique molecular profiles and therapeutic needs.

Previous studies on SNSCC have mainly focused on genetic mutations and environmental factors influencing cancer development. Fewer studies have explored expression patterns, which are crucial for understanding the underlying carcinogenic pathways and potential targeted therapies. However, the SNSCC carcinogenesis pathway and targeted therapeutics have not been fully elucidated.

To address this gap, our study integrated proteomic and bioinformatic analyses to identify the key molecular mechanisms and hub proteins involved in SNSCC carcinogenesis. Using molecular docking, we also identified potential repurposable drugs for targeting upregulated hub proteins. These findings help to understand SNSCC carcinogenesis and potential therapeutic targets, which have not been fully elucidated. This research provides the deeper molecular mechanisms of SNSCC and identifies targeted therapeutic strategies.

## 2. Materials and Methods

### 2.1. Proteomic Dataset and SNSCC Patients

Publicly available proteomic datasets were obtained from our cohort study (accession number MSV000095570) [30]. All differentially expressed gene symbols from proteomic analysis were used in the downstream analysis. For the validation experiment, formalin-fixed, paraffin-embedded (FFPE) tissue samples of SNSCC (*n* = 61) and NP (*n* = 54) were retrospectively collected from Srinagarind Hospital, Khon Kaen University, Thailand. Ethical approval was obtained to use reserved FFPE specimens. The study was conducted in accordance with Khon Kaen University Ethics Committee guidelines and with the Declaration of Helsinki in Human Research.

### 2.2. Protein–Protein Interaction (PPI) Network Construction and Identification of Significant Modules and Hub Proteins

To construct the overall potential mechanism of nasal carcinogenesis in the PPI network, functional enrichment analysis of the differentially expressed proteins (DEPs) was performed using Gene Ontology (GO) annotation, specifying biological process (BP) with ClueGO version 2.5.10 (accessed in November 2023) [31], a Cytoscape plug-in. Bonferroni-corrected *p*-values were applied to select enrichment GO terms, and terms with a q-value < 0.05 were considered statistically significant. The PPI network was analyzed with Molecular Complex Detection (MCODE) version 2.0.3 (accessed in November 2023) [32], a Cytoscape plug-in, to identify highly interconnected modules. The stringency parameter was set with a degree cut-off of 5 and an include-loop option enabled. Modules with an MCODE score ≥ 4 and nodes ≥6 were selected for further analysis [33,34]. The biological relevance of each module was assessed using functional enrichment analysis in STRING version 12.0 with FDR < 0.05, focusing on their involvement in carcinogenesis pathways. Hub proteins were identified based on their inclusion in modules with high MCODE scores and their significant enrichment in processes directly related to carcinogenesis, such as histone modification or RNA splicing. These hub proteins were considered potential key regulators in SNSCC.

### 2.3. Relative Gene Expression by qRT-PCR

Total RNA was isolated using a High Pure RNA Paraffin Kit (Roch, Mannheim, Germany) according to the manufacturer’s instructions. Total RNA (1 μg) was used to synthesize cDNA according to the manufacturer’s protocol (RevertAid H minus First Strand cDNA Synthesis Kit, ThermoFisher Scientific, Waltham, MA, USA). The cDNA samples were used to analyze the expression of target genes by real-time PCR on a QuantStudio 6 Flex Real-Time PCR instrument (Applied Biosystems, Foster City, CA, USA) using SsoAdvanced SYBR Green Supermix (Bio-Rad, Hercules, CA, USA). GAPDH was used as an internal control. The 2^−ΔΔCT^ method was used to calculate the relative expression level determined from real-time quantitative PCR experiments. Only targeted genes with CT values < 35 were used to calculate the relative expression level. The primers used in the present study are listed in Table S1.

### 2.4. The Identification of Potential Repurposable Drugs

L1000CDS^2^ and iLINCS web-based search engine applications were employed to identify potentially repurposable drugs (accessed in September 2024) [35,36]. Gene symbols of DEPs in SNSCC were used as input for both approaches. Repurposable drugs were identified based on their negative correlation with DEPs, indicating their potential to inhibit disease-associated gene expression changes. In iLINCS, inhibitors were selected from the chemical perturbagen library using a concordance threshold score of ≤−0.321, an established highly discordant score cutoff related to disease signatures [37]. In L1000CDS^2^, the top 50 chemical perturbagens were selected based on their ability to counteract the expression patterns of DEPs in SNSCC. Drugs identified by both methods were considered as inhibitor candidates for SNSCC treatment.

### 2.5. Identification of Potential Drug Target of SNSCC by Molecular Docking

The 3D structures of 10 inhibitor drug candidates were downloaded from the PubChem database. The online tool SwissADME software (http://www.swissadme.ch, accessed on 14 October 2024) was used to assess the drug-likeness of the compounds according to Lipinski’s rule of five and Veber’s rule [38,39]. The 3D structures of TATA box-binding protein-associated factor 1 (TAF1) and TATA box-binding protein-associated factor 1 with AZD6738 (PDB ID: 7JSP) [40] were obtained from the PDB database (https://www.rcsb.org/, accessed on 15 October 2024). Chimera version 1.18, a software package for visualization, preparation of receptors and ligands, and data analysis, was used to demonstrate the binding of the candidate inhibitors and TAF1 protein. The ligand and receptor were prepared for docking simulations using Dock Prep [41], which included adding hydrogen, assigning charges, and generating molecular surfaces. Chimera with the AutoDock Vina package was utilized for docking simulations between the ligand and receptor. The binding site of TAF1 was centered on AZD6738, and the grid size was set to 25 × 25 × 25 points with the corresponding center coordinates (x = 20.762, y = 18.995, and z = 65.000 A°). The docking pose with the lowest binding energy and minimum root mean square deviation (RMSD) was chosen as the most suitable. Chimera and BIOVIA Discovery Studio Visualizer software were used to elucidate the details of 3D and 2D ligand–receptor interactions, respectively.

### 2.6. Statistical Analysis

Statistical analysis was performed using GraphPad Prism 9 software (GraphPad Software Inc., San Diego, CA, USA). Two-group analysis was performed using nonparametric tests with the Mann–Whitney test. Statistical tests were two-sided. A *p*-value < 0.05 was considered to indicate a statistically significant difference.

## 3. Results

### 3.1. Gene Ontology Annotation and Protein–Protein Interaction (PPI) Network

**Figure 1:**
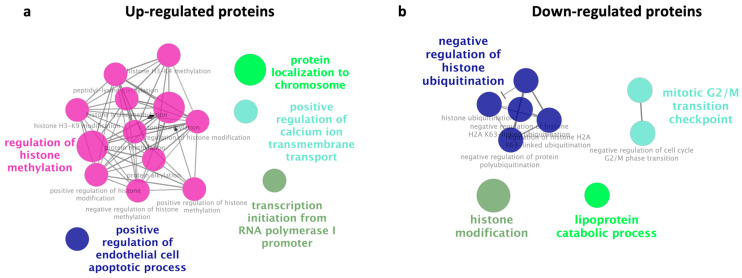
Gene ontology with the biological process network of the differential expression proteins using ClueGO. Statistical FDR < 0.05 was considered statistically significant. Upregulated proteins are mainly linked to histone methylation regulation (indicated by pink dots) (**a**). Downregulated proteins are mainly linked to negative regulation of histone ubiquitination (indicated by blue dots) (**b**). The bold fonts indicate the most important functional GO terms of each group.

To determine the potential mechanism of nasal carcinogenesis, DEPs were analyzed for biological processes using ClueGo with GO (GO: BP). For the upregulated proteins, regulation of histone methylation, positive regulation of endothelial cell apoptotic process, positive regulation of calcium ion transmembrane transport, protein localization to chromosome, and transcription initiation from RNA polymerase I promoter were significantly enriched GO terms (Figure 1a). Conversely, downregulated proteins were involved in the negative regulation of histone ubiquitination, histone modification, the lipoprotein catabolic process, and the mitotic G2/M transition checkpoint (Figure 1b). These results suggest that proteins related to histone modifications may be involved in the development of SNSCC.

**Figure 2:**
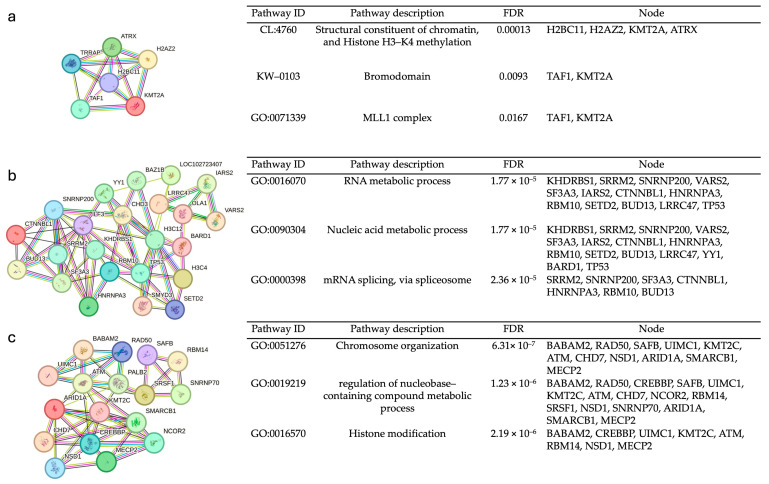
Top three modules of SNSCC by MCODE and enrichment pathways by STRING. Statistical FDR < 0.05 was considered statistically significant: (**a**) Module 1 with upregulated proteins, mainly associated with histone H3-K4 methylation and bromodomain, processes linked to chromatin remodeling and transcriptional regulation. (**b**) Module 2 with downregulated proteins, mainly associated with spliceosome activity, highlighting disruptions in RNA processing mechanisms. (**c**) Module 3 with downregulated proteins, mainly associated with histone modification.

To gain insights into the molecular mechanism of SNSCC carcinogenesis, the modules in the PPI network were determined with the MCODE application of Cytoscape. In the present study, one upregulated module and two downregulated modules were identified with MCODE scores of ≥4. The proteins in each module are listed in Table 1. In addition, pathway enrichment analysis of the hub proteins of each module was performed with the STRING database. Functional annotation with biological processes or pathways was associated with structural constituents of chromatin and histone H3-K4 methylation and bromodomain in module 1, suggesting a critical role in chromatin remodeling and transcriptional regulation (Figure 2a). Modules 2 and 3, downregulated proteins, are linked to RNA processing via spliceosome activity and alterations in epigenetic regulation through histone modification, respectively (Figure 2b,c).

Histone modification regulates gene expression and chromatin structure [42]. Aberrant expression of histone modification can lead to dysregulated transcription of oncogenes or tumor suppressor genes, contributing to SNSCC development. Dysregulation of spliceosome subunits, which are essential for mRNA maturation and gene expression during post-transcriptional modification, is associated with increased DNA damage and cell cycle defects [43]. These results suggest that hub proteins could play a crucial role in SNSCC carcinogenesis through, at least, spliceosome and histone modification dysregulation.

### 3.2. Alteration of Spliceosome Components Involved in SNSCC Carcinogenesis

**Figure 3:**
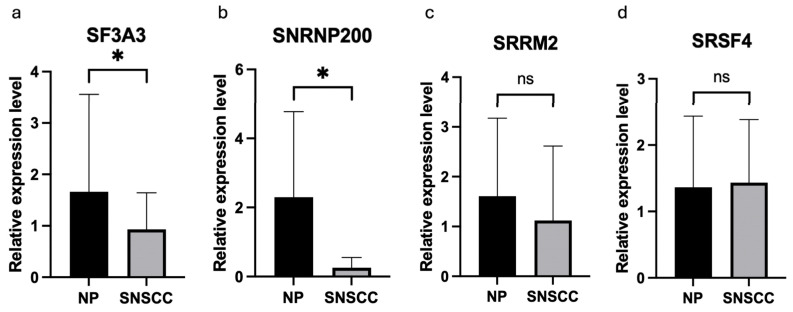
Relative expression of spliceosome-related genes in SNSCC and NP tissues. (**a**) SF3A3, (**b**) SNRNP200, (**c**) SRRM2, and (**d**) SRSF4 levels were analyzed by qRT-PCR. Results are presented as fold change relative to the expression in NP tissues. SF3A3 and SNRNP200 showed significant differences between SNSCC and NP tissues, suggesting dysregulation of these proteins in SNSCC pathogenesis. Statistical significance was calculated using a Mann–Whitney test (*p* < 0.05). * *p* < 0.05, ns = not significant.

Dysregulation of spliceosome subunits, which are important for mRNA splicing and gene expression, is linked to genomic instability, cell cycle progression, and potential cancer transformation [44]. Dysregulation of spliceosomal components can contribute to carcinogenesis by mRNA maturation and pre-mRNA splicing. Our study identified the highest MCODE score associated with mRNA splicing, suggesting that this cluster is highly interconnected. This indicates its potential role as a key regulator in carcinogenesis mechanisms. Therefore, SNRNP200, SF3A3, SRRM2, and SRSF4, involved in mRNA splicing via the spliceosome, were selected for further validation in tissue samples by using RT-qPCR. The characteristics of NP and SNSCC patients are shown in Table S2. The age of the SNSCC group was significantly older than the NP group. The expression levels of SNRNP200 and SF3A3 were significantly decreased in SNSCC tissues compared with those in NP tissues (*p* = 0.0185 and *p* = 0.0235, respectively; Figure 3). However, the expression of SRRM2 and SRSF4 did not show significantly altered mRNA levels in SNSCC tissues compared with those in NP tissues.

The downregulation of SNRNP200 and SF3A3 mRNA may result in aberrant RNA splicing, contributing to the making of oncogenic splice variants or the loss of functional tumor suppressor isoforms. These results suggest that the dysregulation of some proteins in the spliceosome pathway, as evidenced by the downregulation of SNRNP200 and SF3A3 mRNA, may play a critical role in the tumorigenesis of SNSCC.

### 3.3. Potential Repurposable Drug Identification

**Figure 4:**
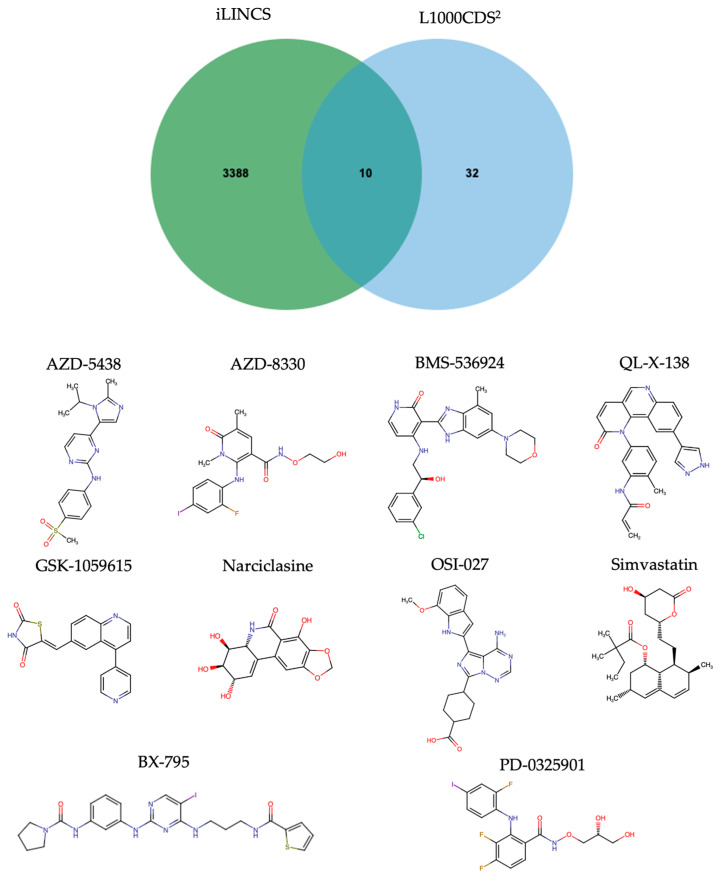
Venn diagram of inhibitors that reverse gene expression in SNSCC identified by L1000CDS^2^ and iLINCS. The 10 common inhibitors and their chemical structures are presented in the lower panel.

The L1000CDS^2^ and iLINCS online databases were used to identify potential drug candidates for SNSCC treatment through a gene expression analysis of drug perturbation experiments. These databases collect host gene expression profiles altered by drug perturbation experiments. Gene symbols of the DEPs in SNSCC were used to identify potential drugs for repurposing that negatively correlated to those of the DEPs. Using this approach, the L1000CDS^2^ platform identified 42 inhibitors, while iLINCS identified 3398 inhibitors (Figure 4). Notably, 10 inhibitors were common to both approaches, suggesting their potential as therapeutic agents for SNSCC.

The perturbation details of the 10 small molecules across different cell lines and doses are listed in Table S3. According to a literature review, all the identified inhibitors exhibited anti-cancer activity via various molecular pathways, indicating their potential as therapeutic agents for SNSCC (Table 2).

### 3.4. OSI-027 Might Target TAF1 by Molecular Docking Studies

Lipinski’s rule of five and Verber’s rules are guidelines used to evaluate the drug-likeness and predict the oral bioavailability of compounds. Using SwissADME predictor, all the candidate inhibitors, except for BX-795, exhibit these desirable drug-like properties (Table 3).

Molecular docking was used to identify the most potent inhibitor and reveal its interaction with the protein target. The technique is one of the most widely used in silico methods in structure-based drug design [29]. In this study, TAF1 was proposed as a promising target for SNSCC therapy due to its (i) central role as an upregulated hub protein in SNSCC, (ii) involvement in transcriptional and histone modification, and (iii) overexpression in several cancer types, as revealed by a literature review. TAF1 is a component of the TFIID complex that plays a central role in initiating transcription by RNA polymerase II and gene expression regulation. TAF1 features a double bromodomain (BD1 and BD2), a protein module that binds to acetylated lysine residues on histones and transcription factors [40]. Therefore, we performed the molecular docking of the co-crystallized TAF1-BD2 receptor (PDB ID: 7JSP) with 10 candidate inhibitors in the active pocket of TAF1. The compounds AZD6738, AZ20, and GNE-371 were used as reference inhibitors. The molecular docking results revealed that four compounds, OSI-027, BMS-536924, Simvastatin, and BX-795, exhibited higher binding affinities than the reference inhibitors (Table 4).

**Figure 5:**
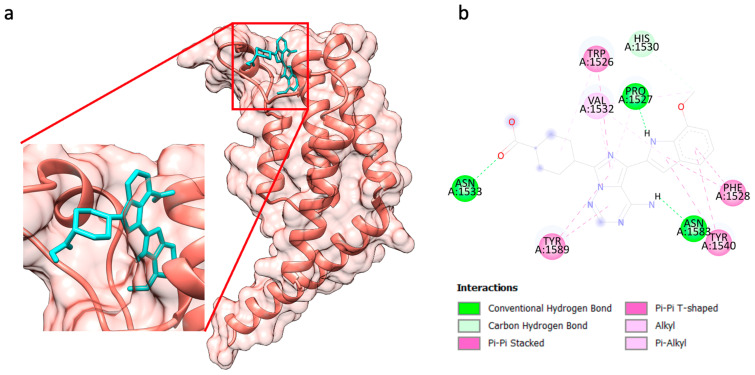
Molecular docking analysis of TAF1 with OSI-027: (**a**) 3D structure of TAF1 (salmon pink) bound to OSI-027 (cyan), the close-up view indicating the active site binding pocket; (**b**) 2D structure of TAF1’s interaction with OSI-027, generated using BIOVIA Discovery Studio Visualizer v24.1.0 software, demonstrating key interactions such as hydrogen bonds, pi-pi stacking, and alkyl, indicating the molecular basis for the strong binding affinity observed in docking analysis.

Previous studies have revealed that TAF1 inhibitors interact with the TAF1 protein by forming H-bonds at ASN1583, with or without ASN1533 and PRO1531 [40]. Notably, OSI-027, BX-795, GSK1059615, narciclasine, and AZD-8330 formed H-bonds with key residues at ASN1583, a critical acetylated lysine interaction site for TAF1 inhibitors (Table S4). OSI-027 exhibited the highest binding affinity (−10.0 kcal/mol), forming hydrogen bonds with ASN1583, ASN1553, and PRO1527 (Table 4 and Figure 5). The additional ligand TAF1 interactions are detailed in Table S4.

Therefore, the highest binding affinities and interactions being at the active site with OSI-027 indicate that this inhibitor may serve as a promising therapeutic drug by targeting the TAF1 protein.

## 4. Discussion

SNSCC is a rare tumor, usually diagnosed at an advanced stage due to non-specific symptoms, with high mortality and recurrence rates. However, the SNSCC carcinogenesis pathway has not been fully elucidated. Therefore, identification of the key molecular mechanisms and hub proteins driving SNSCC is critical for understanding nasal carcinogenesis. Proteomic technologies have enabled analysis of the expression levels of thousands of proteins. The alteration of protein expression between NP and SNSCC may be involved in nasal tumorigenesis. In the present study, the differentially expressed proteins were used to investigate the molecular mechanisms underlying SNSCC carcinogenesis.

Epigenetic modifications are changes in gene expression without alterations to the DNA sequence. DNA methylation and several types of histone modifications (acetylation, methylation, and ubiquitination) play major roles in these processes [42]. The functional enrichment analysis of the upregulated proteins indicated an association with the process of regulation of histone modification, especially histone methylation. This is consistent with previous findings that histone methylation plays a crucial role in head and neck cancer development by post-translational modification in chromatin structure regulation [42,83]. Aberrant histone methylation can lead to both open and condensed chromatin conformations, resulting in gene activation and repression. For instance, H3K4me3 promotes an open chromatin conformation that facilitates gene expression, whereas H3K27me3 promotes chromatin condensation and gene repression. In oral squamous cell carcinoma (OSCC), elevated levels of H3K27me3 are associated with advanced tumor stages and poorer survival rates, with poorer 5-year disease-free survival outcomes [84].

The enrichment analysis of the downregulated proteins identified an association with the process of negative regulation of histone ubiquitination. This process involves the addition or removal of ubiquitin molecules to histone proteins, mainly H2A and H2B, which can either activate or repress gene expression depending on the specific histone and modification site [85,86]. Aberrations in histone ubiquitination and deubiquitination are frequently associated with tumorigenesis [87,88]. In the context of SNSCC, dysregulation of histone methylation and ubiquitination could promote the expression of genes involved in cell proliferation, invasion, and resistance to apoptosis, leading to cancer progression.

Hub proteins play a central role in driving cancer progression. In this study, three modules were identified as potential hub proteins. Hub proteins associated with histone modification and spliceosome dysregulation play a crucial role in SNSCC carcinogenesis. TAF1 is a key component of the transcription factor IID (TFIID) complex, essential for initiating RNA polymerase II-dependent transcription. This protein exhibits diverse functions including kinase, ubiquitin-activating, and histone acetyltransferase (HAT) activities. Using its bromodomains, TAF1 acetylates specific lysine residues on histones H3 and H4, reducing their positive charge on histones and loosening their interaction with negatively charged DNA, leading to the promotion of gene transcription [89,90,91]. This is consistent with previous studies finding that the HAT pathway plays a crucial role in sinonasal undifferentiated carcinoma (SNUC) development [92]. TAF1 overexpression has been reported in various cancers, including leukemia, non-small-cell lung cancer, and glioma, where it serves as an oncogenic driver [93,94,95]. TAF1-dependent histone acetylation facilitated Sp1 binding to the cyclin D1 promoter, leading to transcription and promoting cell cycle progression [96]. Notably, targeting TAF1 has emerged as a therapeutic approach in various cancers [97,98]. Therefore, TAF1 was identified as a therapeutic target in SNSCC based on its upregulated hub protein in module 1. It was found to be overexpressed in several cancer types and involved in transcriptional and histone modification processes. The selective targeting of drugs to TAF1 may be valuable in the treatment of SNSCC. Further functional validations of TAF1 in SNSCC, including in vitro and in vivo studies, are needed to confirm its role in carcinogenesis and explore its potential as a therapeutic target.

Module 2 exhibited the highest MCODE score and was associated with the spliceosome, suggesting that the spliceosome may significantly contribute to SNSCC carcinogenesis. Dysregulation of spliceosome subunits, which are essential for mRNA maturation and gene expression during post-transcriptional modification, is associated with increased DNA damage and cell cycle defects [43]. Splicing alterations can be driven by several mechanisms, including differential regulation during transcription, alternative splicing, nonsense-mediated decay, miRNAs, lncRNAs, and circRNAs [99]. Spliceosome dysregulation has been found in several cancers including pancreatic ductal adenocarcinoma (PDAC), OSCC, and ovarian cancer [100,101,102].

To verify the proposed hub genes, we determined the expression levels of spliceosome-related genes in SNSCC and NP tissues by qRT-PCR. Analysis of potential confounding factors in the comparison between SNSCC and NP indicated that the age of the SNSCC group is significantly older than the NP group. The findings in our data are consistent with those in previous studies, which reported that inflammatory sinonasal polyps predominantly appeared in ages 21–40, while SNSCC is common in ages 53–68 [103,104]. The results showed that SNRNP200 and SF3A3 were significantly downregulated in SNSCC compared to NP tissues. Although SRRM2 and SRSF4 were not significantly different in SNSCC compared to NP tissues, proteomic data showed that these proteins were downregulated in SNSCC. This suggests that post-transcriptional and post-translational mechanisms may regulate SRRM2 and SRSF4 expression in SNSCC.

SNRNP200 is a spliceosomal helicase that unwinds the U4/U6 snRNA. Silencing SNRNP200 in HEK293 cells resulted in differential expressions of genes associated with cell cycle abnormalities [105]. Furthermore, SNRNP200 plays a crucial role in regulating the innate immune response against RNA virus infection [106]. SF3A3, a core U2 snRNP component, is essential for the pre-mRNA splicing process. Numerous studies have shown that the upregulation of SF3A3 could promote oncogenesis by influencing alternative splicing events, such as the activation of oncogenes or inhibition of tumor suppressors [107,108,109]. On the other hand, SF3A3 was downregulated in SNSCC, suggesting that its role may be cancer-type-specific or influenced by post-translational modifications, possibly reflecting distinct molecular pathways involved in different tumor types. To the best of our knowledge, this is the first study to describe the downregulation of SNRNP200 and SRSF4 in SNSCC. Further investigation is needed to elucidate their role in SNSCC. While our study provides significant insights into the dysregulation of spliceosome components, including SNRNP200 and SF3A3 and their potential roles in SNSCC, limitations must be acknowledged. Due to the rarity of SNSCC, we collected SNSCC samples over 10 years at Srinagarind Hospital. The small validation cohort is one limitation of this study. A greater sample size is required in subsequent studies to enhance the robustness and provide deeper insights into the findings.

Advanced SNSCC patients typically undergo multimodal treatment approaches involving surgery and radiotherapy, shown to enhance local disease control and improve the overall survival rate [28]. Chemotherapy may be used as adjuvant or neoadjuvant therapy, with retrospective studies suggesting its potential to increase complete resection and improve prognosis [110]. In this study, using L1000CDS^2^ and iLINCS approaches, we identified 10 candidate inhibitors based on reversing the gene expression changes in SNSCC. Five of the identified candidate drugs are registered for clinical trials (clinicaltrials.gov) as therapies for cancer. These include PD-0325901 (MEK1/2 inhibitor), AZD-8330 (MEK1/2 inhibitor), simvastatin (HMG-CoA inhibitor), OSI-027 (mTORC1/2 inhibitor), and AZD-5438 (CDK1/2/9 inhibitor).

Computational chemistry has become a valuable tool in medicine, and researchers have extensively used in silico methods to study the complex interactions between ligands and receptor targets [29]. The computational approach aims to predict the most binding pose of ligands in the binding pocket of the receptor, resulting in a stable ligand–receptor complex. By analyzing the energetic and geometric complementarity between the ligand and receptor, molecular docking can estimate the binding affinity and stability of the resulting complex, providing crucial insights to guide the development of new drugs and treatments.

Therefore, we linked the 10 candidate repurposable drugs with TAF1 (hub protein) as a novel therapeutic strategy for cancer treatment. The results reveal OSI-027 to be the most promising drug for TAF1-BD2, demonstrating a binding affinity of −10.0 kcal/mol through interactions with key residues at ASN1583. The previous study demonstrated that TAF1 inhibitors (AZD6738, AZ20, and GNE-371) interacted with the TAF1 protein by forming H-bonds at ASN1583 of the lysine-acetylated site [40]. These results suggested that blocking the cancer epigenetic regulator (histone acetylation regulator of TAF1) with OSI-027 may serve as a novel therapeutic strategy for cancer treatment.

OSI-027 is a dual mTORC1 and mTORC2 inhibitor that has been shown to enhance sensitivity to gemcitabine in PDAC and exhibit anti-cancer activity in cholangiocarcinoma and colon cancer [63,64,65]. In early-phase clinical trials, OSI-027 demonstrated a dose-dependent inhibition of mTORC1/mTORC2 activity in patients with advanced solid cancers [111]. However, renal function disturbances were a major kidney-related side effect. Intermittent dosing partially helped reduce the kidney-related side effects, possibly combined with other treatments, which might lower the risk of side effects. In SNSCC, OSI-027 could be applied as a therapeutic option, especially in combination with standard treatments (surgery and radiotherapy), to improve patient outcomes. Moreover, targeting TAF1 could be personalized to patients based on their molecular profiles. Future studies should evaluate the efficacy and mechanisms of OSI-027 action in SNSCC both in vitro and in vivo. Clinical trials could assess the efficacy and safety of OSI-027, both in monotherapy and in combination with standard treatments (surgery and radiotherapy) in SNSCC patients. Additionally, these trials could integrate TAF1 expression as a biomarker to classify patients and optimize therapeutic strategies.

Nevertheless, we used integrated bioinformatics and proteomic analysis to uncover key molecular pathways and identify potential therapeutic targets. We found that OSI-027 may function as an agonist of upregulated hub proteins of TAF1, providing important clues in the development of novel therapeutic strategies for SNSCC.

## 5. Conclusions

Hub proteins and underlying molecular mechanisms in SNSCC carcinogenesis were identified, highlighting histone modification and spliceosome dysregulation as the prominent pathways. Through a drug repurposing approach targeting differentially expressed proteins in SNSCC, 10 candidate drugs were identified, and docking with TAF1 revealed OSI-027 to be the most promising inhibitor of SNSCC. Additional experimental and clinical studies are needed to evaluate the therapeutic efficacy of OSI-027 by itself or in combination with current therapies in SNSCC models.

## Figures and Tables

**Table 1:** PPI network with MCODE scores ≥ 4.

Module	Node	Edge	MCODE Score	Potential Hub Proteins
1	6	14	4.000	H2BC11, H2AZ2, TAF1, KMT2A, ATRX, TRRAP
2	23	62	5.167	SMYD3, BAZ1B, H3C4, KHDRBS1, SRRM2, SNRNP200, VARS2, SF3A3, IARS2, CTNNBL1, HNRNPA3, RBM10, SETD2, BUD13, LRRC47, YY1, ILF3, LOC102723407, BARD1, OLA1, TP53, H3C12, CHD3
3	17	45	5.000	BABAM2, RAD50, CREBBP, SAFB, UIMC1, KMT2C, ATM, CHD7, PALB2, NCOR2, RBM14, SRSF1, NSD1, SNRNP70, ARID1A, SMARCB1, MECP2

**Table 2:** Review of the 10 candidate inhibitors in different types of diseases.

Inhibitor	Action	Molecular Mechanisms	Diseases	**Refs.**
BX-795	PDK1 and TBK1 inhibitor	Anti-cancer activity, enhances sensitivity to chemo and radiotherapy, anti-HSV1 and 2, anti-inflammation	Oral squamous cell carcinoma, neuroblastoma, bladder cancer, HSV-1 and 2 infection, and inflammatory responses triggered by Gram-positive bacteria	[45,46,47,48,49]
PD0325901	MEK1/2 inhibitor	Anti-cancer activity	Papillary thyroid carcinomas, head and neck cancer, colorectal cancer, non-small-cell lung cancer, and pancreatic cancer	[50,51,52,53]
AZD-8330	MEK1/2 inhibitor	Anti-cancer activity and anti-influenza A	Advanced malignancies, non-small-cell lung cancer, and influenza A virus infection	[54,55,56]
Simvastatin	HMG-CoA inhibitor	Reduced low-density lipoprotein (LDL), triglyceride (TG), anti-cancer activity, and enhances sensitivity to chemotherapy	Hyperlipidemia, osteosarcoma, and prostate cancer, bladder cancer and breast cancers	[57,58,59,60,61]
OSI-027	mTORC1/2 inhibitor	Enhances sensitivity to chemotherapy and anti-cancer activity	Pancreatic and colon cancers, and cholangiocarcinoma	[62,63,64,65]
BMS-536924	IGF-IR and IR inhibitor	Anti-cancer activity	Breast, ovarian, glioma, biliary tract, and small-cell lung cancers	[66,67,68,69,70]
GSK-1059615	PI3K and mTOR inhibitor	Anti-cancer activity	Gastric cancer and head and neck squamous cell carcinoma	[71,72]
Narciclasine	Modulates the Rho/Rho kinase/LIM kinase/cofilin signaling pathway and topoisomerase I inhibitor	Anti-cancer activity	Oral, breast, gastric, and esophageal cancers and primary effusion lymphoma	[73,74,75,76,77]
AZD-5438	CDK1/2/9 inhibitor	Anti-cancer activity, enhances sensitivity to radiotherapy, neuroprotective agent	Colorectal, prostate, ovarian, and breast cancers, non-small-cell lung carcinoma, and neurodegenerative diseases	[78,79,80,81]
QL-X-138	BTK/MNK dual kinase inhibitor	Anti-cancer activity	B-cell malignancies	[82]

**Table 3:** Drug-likeness prediction of candidate inhibitors, analysis by SwissADME.

Compound	PubChem CID	Lipinski’s Rule of Five and Veber’s Rule						
		MW	LogP	HBD	HBA	RB	TPA	Drug-Likeness
		<500	<5	<5	<10	<10	<140 Å^2^	
AZD-5438	16747683	371.46	2.51	1	5	5	98.15	yes
AZD-8330	16666708	461.23	2.39	3	5	7	92.59	yes
BMS-536924	135440466	479.96	3.02	4	4	6	106.27	yes
BX-795	10077147	591.47	3.84	4	4	12	139.52	no
GSK1059615	23582824	333.36	2.65	1	4	2	97.25	yes
Narciclasine	72376	307.26	−0.61	5	7	0	128.48	yes
OSI-027	135398516	406.44	2.23	3	6	4	131.42	yes
PD0325901	9826528	482.19	3.18	4	7	8	90.82	yes
QL-X-138	73707530	421.45	3.42	2	4	5	92.67	yes
Simvastatin	54454	418.57	4.11	1	5	7	72.83	yes

MW, molecular weight; HBD, H-bond donors; HBA, H-bond acceptors; RB, rotatable bonds; TPA, topological polar surface area.

**Table 4:** Molecular docking scores (kcal/mol) of selected ligands against TAF1 based on the best protein-ligand binding pose. The table lists the binding affinity of each ligand, with lower values indicating stronger binding interactions.

Ligands	Binding Affinity (kcal/mol)
OSI-027	−10.0
BMS-536924	−8.7
Simvastatin	−8.6
BX-795	−8.5
AZD-6738	−8.2
GNE-371	−8.2
GSK1059615	−8.0
AZD-5438	−7.7
AZ20	−7.4
QL-X-138	−7.3
Narciclasine	−7.3
AZD-8330	−7.1
PD0325901	−7.0

## Data Availability

Raw data are available upon request to the corresponding author.

## Supplementary Materials

The following supporting information can be downloaded at: https://www.mdpi.com/article/10.3390/biology13121089/s1, Table S1: List of primers; Table S2: Characteristics of patients with SNSCC and NP; Table S3: The 10 small molecules’ perturbation details in different cell lines and doses from L1000CDS^2^ and iLINCS; Table S4: Summary of 2D ligand-TAF1 interactions analysis by BIOVIA Discovery Studio Visualizer software.
