# Supplementary material for: OSI-027 as a Potential Drug Candidate Targeting Upregulated Hub Protein TAF1 in Potential Mechanism of Sinonasal Squamous Cell Carcinoma: Insights from Proteomics and Molecular Docking
Source: Biology (Basel). 2024 Dec 23;13(12):1089. doi: 10.3390/biology13121089 (PMC11673211; doi:10.3390/biology13121089)
Supplement: Supplementary file 1 [file biology-13-01089-s001.zip › biology-3344129-supplementary.pdf]

Supplementary Materials

Table S1. List of primers.

Gene	Forward (5′-3′)	Reverse (5′-3′)
GAPDH	TCATCAGCAATGCCTCCTGCA	TGGGTGGCAGTGATGGCA
SNRNP200	CATCGAGGACGAGATGGACG	GCACCTTGGTCTTGGCATTG
SRRM2	GAAGAAAGGCCTGCTGTGTC	CCAAAGCTGTTCTCCCTGAG
SRSF4	GGGAAGGTCGAGGAGAGAGT	CTGGAGCGTGATTCTGATGGA
SF3A3	GGAGAATGGGACCTTTCCTGGA	CTGTCCAAACCCAGAGAAGCCA

Table S2. Characteristics of patients with SNSCC and NP.

Characteristics	NP (n = 54)	SNSCC (n = 61)	*p*-Value	Statistical Tests
**Age (years)**				
< 50	30 (55.5%)	17 (27.9%)		
≥ 50	23 (42.6%)	44 (72.1%)	0.002	Fisher exact test
No clinical data	1 (1.9%)			
**Sex (%)**				
Male	32 (59.2%)	42 (68.9%)	0.4318	Fisher exact test
Female	21 (38.9%)	19 (31.1%)		
No clinical data	1 (1.9%)			
**Inflammation (%)**				
Chronic inflammation	22 (40.7%)			
No inflammation	31 (57.4%)			
No clinical data	1 (1.9%)			
**Cell differentiation (%)**				
Undifferentiated		1(1.63)		
Poorly differentiated		4 (6.55)		
Moderately differentiated		10 (16.39)		
Well differentiated		6 (9.83)		
No clinical data		40 (65.57)		

Table S3. The 10 small molecules' perturbation details in different cell lines and doses from L1000CDS² and iLINCS.

L1000CDS²				
**Perturbagen**	**Cell-Line**	**Dose**	**Time**	**Score**
BX-795	PC3	10 µM	24h	0.0258
AZD-8330	A375	1.11µM	24h	0.0258
GSK-1059615	MCF7	10µM	24h	0.0258
OSI-027	HME1	1.11µM	24h	0.0258
QL-X-138	MCF10A	10µM	24h	0.0258
Narciclasine	MCF7	10µM	24h	0.0232
PD-0325901	A375	3.33µM	24h	0.0232
AZD-5438	LNCAP	10µM	24h	0.0232
Simvastatin	A375	10µM	24h	0.0206
BMS-536924	A375	11.1µM	24h	0.0206
iLINCS				

SignatureId	Perturbagen	Cell-line	Dose	Time	Concordance	pValue
LINCSCP_166133	BX-795	SKL	3.33µM	24h	-0.6685499	1.77819E-06
LINCSCP_113400	PD-0325901	HME1	0.37µM	3h	-0.6308583	9.80073E-06
LINCSCP_81157	AZD-8330	A549	0.37µM	24h	-0.5467792	0.000216562
LINCSCP_266251	Simvastatin	PC3	10µM	24h	-0.5254487	0.000419197
LINCSCP_32772	OSI-027	MCF7	10µM	6h	-0.5139705	0.000587619
LINCSCP_30538	BMS-536924	MCF7	10µM	24h	-0.4871587	0.001236892
LINCSCP_8021	GSK-1059615	A549	10µM	6h	-0.4480846	0.003304191
LINCSCP_36241	Narciclasine	MCF7	10µM	24h	-0.4376518	0.004215542
LINCSCP_86357	AZD-5438	BT20	0.04µM	3h	-0.4326646	0.004723688
LINCSCP_49997	QL-X-138	PHH	10µM	24h	-0.3642409	0.019221025

Table S4. Summary of 2D ligand-TAF1 interactions analysis by BIOVIA Discovery Studio Visualizer software. TAF1 inhibitor binding site is identified based on previous studies.

**Inhibitor**	**TAF1 Inhibitor Binding Site**	**Other Interaction**
AZD6738	H-bond<br>ASN1583<br>ASN1533	Pi-Pi T-shaped/Pi-Pi Stacked<br>TRP1526<br>Pi-Sulfur<br>PHE1536<br>Alkyl/Pi-Alkyl<br>TYR1582, VAL1532, TYR1540, VAL1537, TYR1589<br>C-H bond<br>PRO1527
AZ20	H-bond<br>ASN1583<br>C-H bond<br>PRO1531	Pi-Pi T-shaped/Pi-Pi Stacked<br>TRP1526, TYR1586<br>Alkyl/Pi-Alkyl<br>PRO1527, VAL1532, PHE1536, VAL1537
GNE-371	H-bond<br>ASN1583<br>C-H bond<br>PRO1531	Pi-Pi T-shaped/Pi-Pi Stacked<br>TYR1540<br>Pi-Sigma<br>TRP1526<br>Alkyl/Pi-Alkyl<br>PHE1528, VAL1532, VAL1537<br>C-H bond<br>PRO1527, PHE1536
BX-795	H-bond<br>ASN1583	H-bond<br>TRP1526<br>Pi-Pi T-shaped/Pi-Pi Stacked<br>PHE1528<br>Alkyl/Pi-Alkyl<br>TYR1589, TYR1582, VAL1532, VAL1537, HIS1530
PD-0325901	H-bond<br>PRO1531	H-bond<br>PRO1527, HIS1530<br>Pi-Pi T-shaped/Pi-Pi Stacked<br>TRP1526<br>Pi-Sigma<br>VAL1532<br>Alkyl/Pi-Alkyl<br>PHE1528, VAL1537, TYR1540
AZD-8330	H-bond<br>ASN1583	H-bond<br>HIS1530

		Pi-Pi T-shaped/Pi-Pi Stacked<br>TYR1589, TRP1526<br>Alkyl/Pi-Alkyl<br>PRO1527, PHE1528, VAL1532
Simvastatin		H-bond<br>TYR1540<br>Alkyl/Pi-Alkyl<br>PHE1536, VAL1537, TYR1589, VAL1532, TYR1582
OSI-027	H-bond<br>ASN1583, ASN1533	H-bond<br>PRO1527<br>Pi-Pi T-shaped/Pi-Pi Stacked<br>TRP1526, TYR1589, TYR1540, PHE1528<br>Alkyl/Pi-Alkyl<br>VAL1532<br>C-H bond<br>HIS1530
BMS-536924		Pi-Pi T-shaped/Pi-Pi Stacked<br>TYR1589, TYR1540, PHE1528<br>Pi-Sigma<br>TRP1526<br>Alkyl/Pi-Alkyl<br>VAL1537, PHE1536
GSK-1059615	H-bond<br>ASN1533<br>C-H bond<br>ASN1583	Pi-Pi T-shaped/Pi-Pi Stacked<br>TYR1589<br>Pi-Sigma<br>VAL1537<br>Pi-Sulfur<br>TRP1526<br>Alkyl/Pi-Alkyl<br>VAL1532<br>C-H bond<br>HIS1530
Narciclasine	H-bond<br>ASN1583	Pi-Pi T-shaped/Pi-Pi Stacked<br>TYR1589<br>Alkyl/Pi-Alkyl<br>PRO1527, PHE1528, VAL1532, TYR1540
AZD-5438		H-bond<br>TYR1540<br>Pi-Pi T-shaped/Pi-Pi Stacked<br>TYR1589<br>Pi-Sigma<br>VAL1537<br>Alkyl/Pi-Alkyl<br>VAL1532, TRP1526
QL-X-138		Pi-Pi T-shaped/Pi-Pi Stacked<br>TYR1589, TRP1526, PHE1536<br>Alkyl/Pi-Alkyl<br>VAL1537, VAL1532
